# A Monocentric Cohort of Obstetric Seronegative Anti-Phospholipid Syndrome

**DOI:** 10.3389/fimmu.2018.01678

**Published:** 2018-07-20

**Authors:** Simona Truglia, Antonella Capozzi, Silvia Mancuso, Serena Recalchi, Francesca Romana Spinelli, Carlo Perricone, Caterina De Carolis, Valeria Manganelli, Gloria Riitano, Tina Garofalo, Agostina Longo, Sara De Carolis, Cristiano Alessandri, Roberta Misasi, Guido Valesini, Maurizio Sorice, Fabrizio Conti

**Affiliations:** ^1^Reumatologia, Dipartimento di Medicina Interna e Specialità Mediche, Sapienza Università Roma, Rome, Italy; ^2^Dipartimento di Medicina Sperimentale, Sapienza Università di Roma, Rome, Italy; ^3^Centro Polimedico per la Prevenzione dell’Aborto Spontaneo Ricorrente, Rome, Italy; ^4^Dipartimento di Ginecologia e Ostetricia, Università Cattolica del Sacro Cuore, Rome, Italy

**Keywords:** anti-phospholipid syndrome, seronegative anti-phospholipid syndrome, anti-vimentin/cardiolipin, thin-layer chromatography, anti-phosphatidylserine/prothrombin

## Abstract

The present study was conducted to diagnose obstetric anti-phospholipid syndrome (OAPS) in patients with clinical signs suggestive of anti-phospholipid syndrome (APS), but persistently negative for conventional anti-phospholipid antibodies (aPL). Sera from 61 obstetrical seronegative APS (SN-APS) patients were analyzed for anti-cardiolipin antibodies (aCL) using thin-layer chromatography (TLC)-immunostaining, for anti-cardiolipin/vimentin antibodies (aCL/Vim), anti-phosphatidylserine/prothrombin antibodies, IgA anti-β_2_glycoprotein I antibodies (aβ_2_GPI), and IgA aCL antibodies by enzyme-linked immunosorbent assay. Taken together, our findings show that in 50 out of 61 SN-APS (81.9%) at least one aPL/cofactor antibody was detected using the assays under test. Results revealed that 76% of SN-APS patients resulted positive for aCL by TLC-immunostaining, 54% for aCL/Vim, 12% for aPS/PT, 4% for IgA aβ_2_GPI, and 2% for IgA aCL. Thirty-five out of 61 patients were followed up and the tests were repeated on two occasions, at least 12 weeks apart. Twenty-six out of 35 SN-APS (74.3%) were positive at least one non-conventional test; only 2 patients (5.7%) did not confirm the positivity to the second test. These findings suggest that non-conventional tests, mainly aCL/Vim and aCL detected by TLC-immunostaining, seem to be the most sensitive approaches for finding out aPL in patients with obstetric SN-APS. The use of these tests can be useful for accurate and timely diagnosis of patients with obstetrical APS who are negative for conventional laboratory criteria markers.

## Introduction

Anti-phospholipid syndrome (APS) is a systemic autoimmune disease characterized by arterial and venous thrombosis and pregnancy morbidity associated with circulating anti-phospholipid antibodies (aPL) (1). Obstetrical APS (OAPS) is characterized by early recurrent miscarriage, unexplained fetal loss, and/or premature birth due to eclampsia, preeclampsia, or placental insufficiency as stated in the classification criteria for definite APS (2). APS is the most frequently acquired risk factor for a treatable cause of recurrent pregnancy loss (3) and it increases the risk for pregnancy complications associated with placental dysfunction, such as stillbirth, placental abruption, and fetal growth restriction (4). Classification of OAPS requires the combination of at least one clinical and one laboratory criterion (2), including anti-cardiolipin (aCL) and anti-β_2_glycoprotein I (aβ_2_GPI) antibodies detected by enzyme-linked immunosorbent assay (ELISA) and the lupus anticoagulant (LA) detected by clotting assays (1). In clinical practice there are individuals with clinical signs highly suggestive of APS who are persistently negative for conventional aPL laboratory tests; therefore, physicians proposed for this population the term of “seronegative APS” (SN-APS) (5, 6). Since APS is the commonest treatable cause of recurrent miscarriage, for women with a history of recurrent early abortions or fetal loss, a diagnosis of APS significantly improves the rate of live births (7). New antigenic targets or methodological approaches to detect aPL in SN-APS have been investigated and several non-conventional anti-phospholipid antibodies have been described (8, 9). Recently, with a proteomic approach, we identified cardiolipin/vimentin (CL/Vim) as a “new” target for APS, also detectable in SN-APS patients (10). In addition, it has demonstrated the possibility of detecting aPL in SN-APS patients by immunostaining on thin-layer chromatography (TLC) plates (11, 12).

The aim of the present study was to investigate the potential clinical usefulness of “new” antigenic targets and methodological approaches in detecting serum aPL in patients with obstetrical SN-APS (OSN-APS).

## Materials and Methods

### Patients

The study includes all consecutive patients, presenting clinical features consistent with a diagnosis of obstetrical APS but tested persistently negative for conventional aCL, aβ_2_GPI, and LA tests referred to the Lupus Clinic, Rheumatology Unit of the Sapienza University of Rome from 2012 to 2017. Clinical manifestations consisted of pregnancy morbidity in women with and without history of thrombosis as stated in the classification criteria for definite APS (2).

Sera were collected at several times and stored at −20°C until use. Moreover, all patients were tested for common inherited thrombophilic defects, such as protein C and protein S deficiency, hyperhomocysteinemia, factor V Leiden, MTHFR, and prothrombin mutations. This study was approved by the local ethic committees and participants gave written informed consent.

### ELISA for Anti-Cardiolipin and Anti-β_2_Glycoprotein I Antibodies

Antibodies specific for aCL and aβ_2_GPI (IgG, IgM, and IgA) were detected by ELISA, using QUANTA Lite™ detection kit (INOVA Diagnostic Inc., San Diego, CA, USA) assay. ELISA was performed for all the patients’ sera according to manufacturer’s instructions; a positive control and several normal human sera were run in the same assay to confirm the specificity of the results.

### Chemiluminescence Assay

IgG, IgM, and IgA for aCL and aβ_2_GPI were also tested by chemiluminescence assay using Zenit RA Immunoanalyzer (A. Menarini Diagnostics, Florence, Italy).

### LA Test

Lupus anticoagulant was studied in two coagulation systems, a dilute sensitized activated partial thromboplastin time and a dilute Russell’s viper venom time, followed by confirm test, using reagents and instrumentation by Hemoliance Instrumentation Laboratory, Lexington, MA, USA.

### Detection of aCL by TLC-Immunostaining

Thin-layer chromatography-immunostaining was performed as previously described, with slight modification (11, 12). Briefly, 2 µg (in chloroform/methanol, 2:1 v/v) of cardiolipin (CL, Sigma-Aldrich) were run on aluminum-backed silica gel 60 (20 × 20) high-performance thin-layer chromatography plates (Merck Co., Darmastdt, Germany). Chromatography was performed in chloroform:methanol:CH_3_COOH:water (100:75:7:4) (v:v:v:v) as eluent system. The dried chromatograms were soaked for 90 s in a 0.5% (w:v) solution of poly(isobutyl methacrylate) beads (Polysciences, Eppelheim, Germany) dissolved in hexane. After air-drying for 5 min, the chromatograms were incubated at room temperature for 1 h with 1% bovine serum albumin (BSA) in phosphate buffered saline (PBS) (blocking buffer) to eliminate non-specific binding. After washing by gentle shaking 3 times for 10 min in PBS containing 0.1% Tween 20 (PBS-T), the chromatograms were incubated with sera diluted 1:100 in the blocking solution, for 1 h at room temperature. Sera were removed and chromatograms were washed as above. Bound antibodies were visualized with horseradish peroxidase (HRP)-conjugated goat anti-human IgG (Sigma-Aldrich) diluted 1:1,000 in blocking buffer and incubated at room temperature for 1 h. After washing, immunoreactivity was assessed by chemiluminescence reaction using the enhanced chemiluminescence Western blotting system (Amersham Pharmacia Biotech, Buckinghamshire, UK).

### Detection of Anti-Cardiolipin/Vimentin Complex (aCL/Vim) Antibodies by ELISA

Anti-cardiolipin/vimentin complex antibodies were detected by ELISA with slight modification of previously reported method (13). Ninety-six-well polystyrene plate (Thermo Fisher Scientific, Waltham, MA, USA) were coated and incubated overnight at 4°C with 100 μl/well of CL (from bovine heart, Sigma-Aldrich, Milan, Italy) (50 µg/ml in methanol), and then with 100 μl/well of human recombinant vimentin (5 µg/ml in 0.05 mM NaHCO_3_ buffer, pH 9.5) (R&D System, Minneapolis, MN, USA). Coated plates were incubated overnight at 4°C and then washed three times with PBS-T. Plates were blocked with 100 µl of 1% BSA in PBS (blocking buffer) for 2 h at room temperature. After three washes with PBS-T the wells were incubated with 100 µl of patients sera (diluted 1:100 in the blocking buffer) for 1 h at room temperature. Goat polyclonal anti-vimentin (R&D Systems) was used as positive control. After washing, as above, the plates were incubated for 1 h at room temperature with HRP-conjugated antibodies, either goat anti-human IgG or rabbit anti-goat IgG (Sigma-Aldrich), diluted in blocking buffer. The plates were washed three times with PBS-T, the bound peroxidase was then revealed with *O*-phenylenediamine dihydrochloride development buffer (100 μl/well) and stopped with 50 μl/well of H_2_SO_4_ 0.2 M for 5 min. Absorbance was measured at 492 nm in a microplate reader. ELISA assay was also performed without coated vimentin/cardiolipin complex. Virtually no reactivity was detected in all the samples (data not shown).

Data were analyzed as the mean optical density (OD) corrected for background (wells without coated antigen). Thirty-two normal human sera were also tested and a cutoff value was established as mean of OD ± 3 SDs of normal human sera. Each serum was analyzed in triplicate.

### Detection of Anti-Phosphatidylserine/Prothrombin (aPS/PT) Antibodies by ELISA

Antibodies specific for aPS/PT were detected by ELISA using a QUANTA Lite™ detection kit (INOVA Diagnostic Inc.). All patient samples including those from healthy donors were tested and ELISA was performed according to manufacturer’s instructions.

### Statistical Analysis

All the statistical analyses were performed by GraphPad Prism software Inc. (San Diego, CA, USA). Kolmogorov–Smirnov test was used to assess the normal distribution of the data. Differences between numerical variables were tested with the Wilcoxon test. Statistical coefficient Cohen’s kappa was used to analyze agreement between first and second test. For comparison of categorical variables or percentages we used Fisher’s exact and *X*^2^ tests when appropriate. *P*-values less than 0.05 were considered as significant.

## Results

We enrolled 61 women with clinical features consistent with a diagnosis of obstetrical APS, but tested persistently negative for conventional aCL, aβ_2_GPI (detected by both ELISA and Chemiluminescence assay), and LA tests. All OSN-APS patients were Caucasian with a median age of 39 years (IQR 8). The clinical characteristics of patients are reported in Table 1. Mixed thrombotic and obstetrical features were present in 9 patients out of 61 (14.6%) and isolated obstetrical features in remaining patients (85.4%). All patients were tested for common inherited thrombophilic defects: 18/61 (29.5%) patients presented mutation of MTHFR in heterozygosity, with normal levels of homocysteine; 6/61 (9.8%) mutation of MTHFR in homozygosity, with normal levels of homocysteine; 1/61 (1.6%) mutation of V factor of Leiden in homozygosity; 1/61 (1.6%) protein S deficiency. Because of normal level of homocysteine, these patients cannot be considered at increased risk for inherited thrombophilia.

**Table 1:** Clinical characteristics of patients studied.

Characteristics *n* (%)	Obstetric seronegative anti-phospholipid syndrome (OSN-APS) (total) (*n* = 61)	OSN-APS (followed up) (*n* = 35)
Other autoimmune diseases		
Systemic lupus erythematosus	7 (11.5)	5 (14.3)
Discoid lupus erythematosus	5 (8.2)	5 (14.3)
Autoimmune thyroiditis	11 (18.0)	10 (28.6)
Mixed connective tissue disease	3 (4.9)	1 (2.8)
Undifferentiated connective tissue disease	2 (3.3)	1 (28)
Pregnancy morbidity		
Spontaneous abortions^a^	41 (67.2)	25 (71.4)
Intrauterine death of a normal fetus^b^	27 (44.3)	15 (42.9)
Premature births^c^	4 (6.6)	2 (5.7)
Vascular thrombosis^d^	9 (14.6)	6 (17.1)
Arterial thrombosis	4 (6.6)	3 (8.6)
Venous thrombosis	6 (9.8)	4 (11.4)
Recurrent thrombosis	4 (6.6)	3 (8.6)
Non-criteria APS features		
Livedo reticularis	11 (18.0)	8 (22.9)
Thrombocytopenia	4 (6.6)	4 (11.4)
Migraine	9 (14.6)	5 (14.3)
Seizures	1 (1.6)	1 (2.9)
Thrombotic risk factors		
Hypercholesterolemia	3 (4.9)	3 (8.5)
Smoking	10 (16.4)	7 (20.0)
Hypertension	7 (11.5)	6 (17.1)
Oral contraceptive/hormone replacement therapy	1 (1.6)	0 (0)

*^a^3 or + losses <10 weeks of gestation*.

*^b^1 or + losses ≥10 weeks of gestation*.

*^c^Preterm birth <34 weeks due to eclampsia, pre-eclampsia, or placental insufficiency*.

*^d^Thrombosis (arterial, venous, or in small vessels) in any tissue, confirmed by imaging or histopathology (thrombosis without significant inflammation)*.

**Figure 1:**
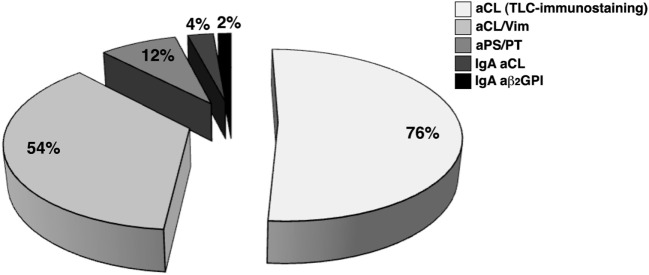
The prevalence of autoantibodies in obstetrical seronegative anti-phospholipid syndrome patients. Fifty out of 61 patients (82%) were positive for at least one of the tests used. Thirty-eight out of 50 patients (76%) showed the presence of anti-cardiolipin (aCL) antibodies detected by thin-layer chromatography-immunostaining, 27/50 (54%) were positive for anti-cardiolipin/vimentin (aCL/Vim) antibodies, 6/50 (12%) for anti-phosphatidylserine/prothrombin antibodies, 2/50 (4%) for IgA aCL, and 1/50 (2%) for IgA aβ_2_GPI.

**Figure 2:**
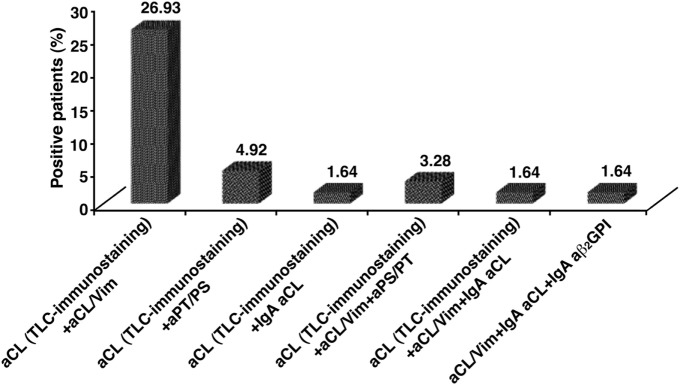
The percentage of obstetrical seronegative anti-phospholipid syndrome patients positive for more than one test used to detect the presence of autoantibodies. The combination of thin-layer chromatography (TLC)-immunostaining and anti-cardiolipin/vimentin (aCL/Vim) approaches detected autoantibodies in the majority of the patients.

Taken together, our findings show that in 50 out of 61 OSN-APS (81.9%) at least one aPL/cofactor antibody was detected using the assays under test; in particular, 50 out of 61 patients (82%) were positive for at least one of the tests used. Thirty-eight out of 50 patients (76%) showed the presence of anti-cardiolipin (aCL) antibodies detected by TLC-immunostaining, 27/50 (54%) were positive for aCL/Vim antibodies, 6/50 (12%) for aPS/PT antibodies, 2/50 (4%) for IgA aCL, and 1/50 (2%) for IgA aβ_2_GPI (Figure 1). Figure 2 shows the percentage of patients displaying multiple positivity for different antibodies. The combination of TLC-immunostaining and aCL/Vim approaches detected autoantibodies in the majority of the patients. No patients were contemporary positive for aCL (by TLC-immunostaining) and aβ_2_GPI IgA antibodies. Table 2 shows autoantibody prevalence in OSN-APS patients according to the clinical manifestations.

**Table 2:** Autoantibody prevalence according to the clinical manifestations.

Autoantibodies (assay) *n* (%)	Arterial or venous thrombosis (*n* = 9)	Pregnancy morbidity (*n* = 61)	Spontaneous abortions (*n* = 41)	Intrauterine death of a normal fetus (*n* = 27)	Premature births (*n* = 4)
Anti-anti-CL [by thin-layer chromatography (TLC)-immunostaining]	7 (77.8)	38 (62.2)	25 (41.0)	18 (29.5)	3 (4.9)
Anti-CL/Vim	4 (44.4)	27 (44.3)	15 (24.6)	13 (21.3)	1 (1.6)
Anti-PS/PT	1 (11.1)	6 (9.8)	3 (4.9)	3 (4.9)	0
Anti-CL IgA	0	2 (3.3)	2 (3.3)	0	0
Anti-β_2_GPI IgA	0	1 (1.6)	1 (1.6)	0	0
Anti-CL (by TLC-immunostaining) + anti-CL/Vim	3 (33.3)	16 (26.2)	11 (18.0)	8 (13.1)	1 (1.6)
Anti-CL (by TLC-immunostaining) + anti-CL/Vim + anti-PS/PT	0	2 (3.3)	2 (3.3)	0	0
No autoantibodies	1 (11.1)	11 (18.0)	10 (16.4)	3 (4.9)	1 (1.6)

*CL, cardiolipin; CL/Vim, cardiolipin/vimentin; PS/PT, phosphatidylserine/prothrombin; β_2_GPI, beta2Glycoprotein I*.

**Figure 3:**
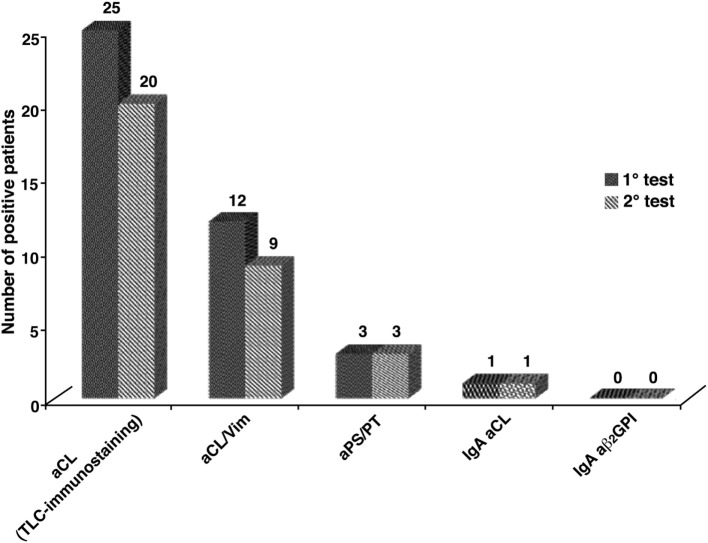
The concordance of the autoantibodies positivity, between first and second test, in obstetrical seronegative anti-phospholipid syndrome followed up where the tests were repeated on two occasions. For thin-layer chromatography (TLC)-immunostaining and anti-cardiolipin/vimentin (aCL/Vim) a “substantial agreement” was found between first and second test, revealed by Cohen’s kappa test (*K* = 0.696 and *K* = 0.789).

Thirty five out of 61 patients were followed up and the tests were repeated on two occasions, at least 12 weeks apart (Table 1). To concern inherited thrombophilic defects: 12/35 (34.3%) patients presented mutation of MTHFR in heterozygosity, with normal levels of homocysteine; 2/35 (0.6%) mutation of MTHFR in homozygosity, with normal levels of homocysteine; 1/35 (0.3%) protein S deficiency. In 26 out of 35 SN-APS (74.3%) at least one aPL/cofactor antibody was detected using the assays under test; only 2 patients (5.7%) did not confirm the positivity in the second test. Figure 3 shows the concordance of the antibodies positivity between first and second test. To concern TLC-immunostaining and aCL/Vim, Cohen’s kappa test resulted respectively of *K* = 0.696 and *K* = 0.789, an agreement that can be described as “substantial agreement” between first and second test.

In this group of patients, a statistically significant correlation was found between anti-PS/PT and anti-CL/Vim (*p* = 0.01), between positivity for aCL (by TLC-immunostaining) and mutation of MTHFR in heterozygosis (*p* = 0.026) and finally between arterial thrombosis and premature births (*p* = 0.039).

The combination of two of the used methodological approaches, TLC-immunostaining for aCL and ELISA for anti-CL/Vim complex antibodies, was able to detect aPL/cofactors in about two-thirds of OSN-APS patients with a small additional gain when also performing ELISA for aPS/PT or aCL and aβ_2_GPI IgA.

## Discussion

In this study new antigenic targets and methodological approaches were used to detect anti-phospholipid antibodies in a monocentric cohort of patients with suspected “seronegative” obstetric APS. Using these approaches, it was possible to demonstrate the presence of aPL in about two-thirds of the enrolled patients.

Differences were not observed in the prevalence of obstetric events, including early spontaneous abortions, fetal deaths, prematurity, or pre-eclampsia, between women with SN-APS and seropositive APS (SP-APS) (14). Likewise, since APS is now recognized as the most common treatable cause of recurrent miscarriage, for women with a history of recurrent early abortions or fetal loss, a diagnosis of APS addresses them toward treatments, which significantly improve the rate of live births (15).

Furthermore, it is mandatory to identify among the so-called SN-APS patients who need long-term secondary thromboprophylaxis (16).

In this regard, we judge not sufficiently satisfying the current panel test to detect antibodies in patients for whom there is a clinical suspicion. This may depend on the limitations of traditional technical approaches or on the existence of antigenic targets other than those known. In order to overcome the limits imposed by conventional tests, we employed a different methodological approach for detection of aCL, TLC-immunostaining, showing the presence of aCL in more than three-quarters (76%) of patients with obstetric SN-APS; these data confirmed the previous results obtained in patients with different records of SN-APS, but also in a case of catastrophic APS (15, 17). This test takes advantage from the different characteristics of binding of phospholipid to solid phase which involves both electrostatic and hydrophobic interactions. Thus, antigen exposure is quite different as compared to that on the surface of microtiter wells, where phospholipids are coated in a layer of immobilized lamellar phospholipids. Our results suggest that this test may represent a very useful tool to detect aPL in the majority of obstetric SN-APS, according to previous papers (11, 18).

Moreover, aPL represents a very heterogeneous family of antibodies and more than 30 different antibodies have been reported in APS patients, the so-called autoantibody explosion in APS (19). Several studies reported various non-conventional aPL in patients with thrombosis and pregnancy morbidity, but relatively few data are available yet. In a study of Zohoury et al., non-criteria tests were used in a cohort of SN-APS and SP-APS showing that anti-CL/Vim antibodies together with aPS/PT were the most sensitive of the non-criteria biomarker in the SN-APS group (20). The present study confirms and extends these data revealing that aCL/Vim antibodies are present in 54% of OSN-APS patients, with a prevalence significantly higher as compared to aPS/PT (12%) in this specific group of SN-APS patients. However, this finding is not surprising, since Žigon et al., who studied the prevalence and clinical association of aPS/PT in patients with a history of pregnancy complications relevant to APS, showed positive of aPS/PT in about 13% of OAPS patients; aPS/PT were the only antibodies associated with early recurrent pregnancy loss, as well as with late pregnancy morbidity and prematurity (21). Indeed, aPS/PT IgG and IgM were shown more frequent in SP-APS than in SN-APS (63 and 37% versus 4 and 5%, respectively) (22).

Taken together, these findings indicate that the execution of all these tests (TLC-immunostaining, aCL/Vim, and aPS/PT) can be very useful for identification of autoantibodies in obstetric SN-APS. The analysis of multiple positivity for different antibodies revealed that the combination of positivity by TLC-immunostaining and aCL/Vim IgG detects aPL in a large proportion of patients. As expected, the contribution of IgA aCL and/or aβ_2_GPI is virtually negligible. Although IgA aCL antibodies have been associated with poliabortivity and fetal deaths in women with primary APS, systemic lupus erythematosus, and unexplained recurrent spontaneous abortion (23), in the present study we showed that the IgA isotypes of the aCL is detectable only in 4% of SN-APS patients. Moreover, in our cohort we found that only 2% of SN-APS patients resulted positive for IgA aβ_2_GPI antibodies. Only few studies showed that IgA aβ_2_GPI antibodies were significantly increased in women with pregnancy morbidity (24, 25).

Thus, the data of the present study suggest that TLC-immunostaining and aCL/Vim seem to be the most sensitive tests able to reveal hidden positivity and, therefore, reducing the risk of a missed diagnosis. The use of new diagnostic tests and new biomarkers will be helpful for clinicians in the accurate and timely diagnosis of patients with obstetrical APS who are negative for conventional laboratory criteria markers. At the end, testing for these antibodies may contribute to the evaluation of the stratification of risk for thrombotic events and/or pregnancy morbidity. In particular, we suggest that in subjects with obstetric APS, the presence of these antibodies may represent an alarm signal. This is important because patients with obstetric SN-APS as well as obstetric SP-APS should be closely monitored by a multidisciplinary team to receive full treatment to have a successful pregnancy outcome.

However, a percentage of obstetric SN-APS remains seronegative to all these tests, indicating that other unidentified cofactors may be involved in sera reactivity. Further studies will shed light on possible “new” antigenic specificities in patients with obstetric SN-APS.

## Ethics Statement

Ethics committee, Sapienza Università di Roma-Policlinico Umberto I. All the patients signed an informed consent prior to enter in the study.

## Author Contributions

FC, MS, GV, and CA conceived and designed the study. AC, SR, VM, and GR performed the experiment. ST, SM, FS, and CP analyzed the data. ST, AC, RM, AL, and TG wrote the original manuscript. FC, MS, RM, CC, and SC read and approved the final version of the manuscript.

## Conflict of Interest Statement

The authors declare that the research was conducted in the absence of any commercial or financial relationships that could be construed as a potential conflict of interest.

## Abbreviations

APS, anti-phospholipid syndrome; aPL, anti-phospholipid antibodies; aCL, anti-cardiolipin; aPS/PT, anti-phosphatidylserine/prothrombin; aCL/Vim, anti-vimentin/cardiolipin; aPTT, activated partial thromboplastin time; aβ_2_GPI, anti-β_2_glycoprotein I; BSA, bovine serum albumin; LA, lupus anticoagulant; dRVVT, dilute Russell’s viper venom time; HRP, horseradish peroxidase; ECL, enhanced chemiluminescence; ELISA, enzyme-linked immunosorbent assay; HPTLC, high-performance thin-layer chromatography; TLC, thin-layer chromatography; OD, optical density; OAPS, obstetrical APS; OSN-APS, obstetrical seronegative APS; PBS, phosphate buffered saline; SLE, systemic lupus erythematosus; SN-APS, “seronegative APS”; SP-APS, seropositive APS; FGR, fetal growth restriction; RSA, recurrent spontaneous abortion.

## References

[1] Hughes GRV The anticardiolipin syndrome. Clin Exp Rheumatol (1985) 3(4):285–6. 4085158

[2] Miyakis S Lockshin MD Atsumi T Branch W Brey RL Cervera R International consensus statement on an update of the classification criteria for definite antiphospholipid syndrome (APS). J Thromb Haemost (2006) 4(2):295–6. 10.1111/j.1538-7836.2006.01753.x 16420554

[3] Branch DW Silver RM Porter TF. Obstetric antiphospholipid syndrome: current uncertainties should guide our way. Lupus (2010) 19(4):446–52. 10.1177/0961203310361490 20353986

[4] Danza A Ruiz-Irastorza G Khamashta MA Antiphospholipid syndrome in obstetrics. Best Pract Res Clin Obstet Gynaecol (2012) 26(1):65–76. 10.1016/j.bpobgyn.2011.10.006 22079775

[5] Hughes GRV Khamashta MA Seronegative antiphospholipid syndrome. Ann Rheum Dis (2003) 62(12):1127 10.1136/ard.2003.006163 14644846 PMC1754381

[6] Nayfe R Uthman I Aoun J Saad Aldin E Merashli Munther M Khamashta MA. Seronegative antiphospholipid syndrome. Rheumatology (Oxford) (2013) 52(8):1358–67. 10.1093/rheumatology/ket126 23502076

[7] Misasi R Capozzi A Longo A Recalchi S Lococo E Alessandri C “New” antigenic targets and methodological approaches for refining laboratory diagnosis of antiphospholipid syndrome. J Immunol Res (2015) 2015:858542 10.1155/2015/858542 25874238 PMC4383493

[8] Valesini G Alessandri C. New facet of antiphospholipid antibodies. Ann N Y Acad Sci (2005) 1051(1):487–97. 10.1196/annals.1361.089 16126989

[9] Sciascia S Amigo MC Roccatello D Khamashta M. Diagnosing antiphospholipid syndrome: ‘extra-criteria’ manifestations and technical advances. Nat Rev Rheumatol (2017) 13(9):548–60. 10.1038/nrrheum.2017.124 28769114

[10] Ortona E Capozzi A Colasanti T Conti F Alessandri C Longo A Vimentin/cardiolipin complex as a new antigenic target of the antiphospholipid syndrome. Blood (2010) 116(16):2960–7. 10.1182/blood-2010-04-279208 20634382

[11] Sorice M Griggi T Circella A Lenti L Arcieri P di Nucci GD Protein S antibodies in acquired proteinS deficiencies. Blood (1994) 83(8):2383–4. 8161807

[12] Conti F Alessandri C Sorice S Capozzi A Longo A Garofalo T Thin-layer chromatography immunostaining in detecting anti-phospholipid antibodies in seronegative. Clin Exp Immunol (2012) 167(3):429–37. 10.1111/j.1365-2249.2011.04532.x 22288586 PMC3374275

[13] Harris EN The second international anti-cardiolipin standardization workshop/The Kaps Group. Am J Clin Pathol (1990) 94(4):476–84. 10.1093/ajcp/94.4.476 2220676

[14] Rodriguez-Garcia JL Bertolaccini ML Cuadrado MJ Sanna G Ateka-Barrutia O Khamashta MA Clinical manifestations of antiphospholipid syndrome (APS) with and without antiphospholipid antibodies (the so-called ‘seronegative APS’). Ann Rheum Dis (2012) 71(2):242–4. 10.1136/annrheumdis-2011-200614 21953349

[15] Conti F Capozzi A Truglia S Lococo E Longo A Misasi R The Mosaic of “Seronegative” Antiphospholipid Syndrome. J Immunol Res (2014) 2014:389601 10.1155/2014/389601 24741593 PMC3987929

[16] Derksen RHWM De Groot PG. Towards evidence-based treatment of thrombotic antiphospholipid syndrome. Lupus (2010) 19(4):470–4. 10.1177/0961203309361483 20353990

[17] Conti F Priori R Alessandri C Misasi R Capozzi A Pendolino M Diagnosis of catastrophic anti-phospholipid syndrome in a patient tested negative for conventional tests. Clin Exp Rheumatol (2017) 35(4):678–80. 28516871

[18] Conti F Alessandri C Spinelli FR Capozzi A Martinelli F Recalchi S TLC immunostaining for detection of "antiphospholipid" antibodies. Methods Mol Biol (2014) 1134:95–101. 10.1007/978-1-4939-0326-9_8 24497357

[19] Schoenfeld Y Twig G Katz U Sherer Y. Autoantibody explosion in antiphospholipid syndrome. J Autoimmun (2008) 30(1–2):74–83. 10.1016/j.jaut.2007.11.011 18171610

[20] Zohoury N Bertolaccini ML Rodriguez-Garcia JL Shums Z Ateka-Barrutia O Sorice M Closing the serological gap in the antiphospholipid syndrome: the value of “Non-criteria” antiphospholipid antibodies. J Rheumatol (2017) 44(11):1597–602. 10.3899/jrheum.170044 28864642

[21] Žigon P Perdan Pirkmajer K Tomšič M Kveder T Božič B Sodin Šemrl S Anti-phosphatidylserine/prothrombin antibodies are associated with adverse pregnancy outcomes. J Immunol Res (2015) 2015:975704. 10.1155/2015/975704 26078985 PMC4452858

[22] Mekinian A Bourrienne M Carbillon L Benbara A Noémie A Chollet-Martin S Non-conventional antiphospholipid antibodies in patients with clinical obstetrical APS: prevalence and treatment efficacy in pregnancies. Semin Arthritis Rheum (2016) 46(2):232–7. 10.1016/j.semarthrit.2016.05.006 27432776

[23] Carmo-Pereira S Bertolaccini ML Escudero-Contreras A Khamashta MA Hughes GRV Value of IgA anticardiolipin and anti-β2-glycoprotein I antibody testing in patients with pregnancy morbidity. Ann Rheum Dis (2003) 62(6):540–3. 10.1136/ard.62.6.540 12759291 PMC1754554

[24] Lee RM Branch DW Silver RM Immunoglobulin A anti-β2-glycoprotein antibodies in women who experience unexplained recurrent spontaneous abortion and unexplained fetal death. Am J Obstet Gynecol (2001) 185(3):748–53. 11568809 10.1067/mob.2001.117659

[25] Yamada H Tsutsumi A Ichikawa K Kato EH Koike T Fujimoto S IgA-class anti-beta2-glycoprotein I in women with unexplained recurrent spontaneous abortion. Arthritis Rheum (1999) 42(12):2727–8. 10.1002/1529-0131(199912)42:12<2727::AID-ANR33>3.0.CO;2-Q 10616025

